# Mental health impact of fentanyl abuse, a case report

**DOI:** 10.1192/j.eurpsy.2024.843

**Published:** 2024-08-27

**Authors:** G. Lorenzo - Chapatte, G. Guerra Valera, P. Marqués Cabezas, L. R. Vázquez, M. Ríos Vaquero, A. Monllor Lazarraga, M. P. Pando Fernández, P. Martínez Gimeno, M. A. Andreo Vidal, M. Calvo Valcárcel, B. Rodríguez Rodríguez, N. Navarro Barriga, M. J. Mateos Sexmero, M. Fernández Lozano, T. Jiménez Aparicio, C. De Andrés-Lobo, M. D. C. Vallecillo Adame, M. D. L. Á. Guillén Soto, L. Sobrino Conde, A. Aparicio Parras

**Affiliations:** Psiquiatría, Sacyl - Hospital Clínico Universitario Valladolid, Valladolid, Spain

## Abstract

**Introduction:**

In recent years, there has been an increase in the prevalence of illicit use of fentanyl and other opioids in the United States population. This has led to an increase in medical, psychopathological and abuse-associated comorbidity, an increase in deaths and a decrease in the age of consumption, and has become a serious emerging problem in young people.

We present the case of an 18-year-old woman from the United States who recently settled in Spain and started a follow-up in Mental Health due to opioid and other substance abuse problems.

**Objectives:**

To address the growing problem surrounding the illicit use of fentanyl and opioids as drugs of abuse based on the presentation of the clinical case mentioned above.

**Methods:**

Bibliographic search and description of a clinical case of a patient under follow-up by Mental Health at the “Hospital Clínico Universitario de Valladolid”.

**Results:**

An 18-year-old woman from the United States who has been living with her father in Spain since the summer of 2023, having moved to Spain due to problems related to substance abuse.

With no previous medical or surgical history and with a history of follow-up in Mental Health in her country of origin for depressive symptomatology, dysfunctional personality traits and abuse of different toxic substances since adolescence.

After a brief and erratic follow-up in Psychiatry for anxious-depressive symptoms reactive to a complex and conflictive relationship with his mother and marked academic difficulties during the first years of adolescence, at the age of 15 he started using cannabis and alcohol, thus beginning a period marked by relationships with marginalized sectors of the population, substance abuse and school failure.

As his cannabis consumption intensified, he began to consume fentanyl prescribed to his mother, as well as other opioids to which he had access illegally, for which reason he had to be admitted twice to detoxification centers without results, which is why his family finally decided to move him to Spain.

**Conclusions:**

In recent years, fentanyl abuse has become a serious public health problem that is mainly centered in the young population.

High levels of impulsivity and lack of frustration tolerance predispose to the use of illicit substances for elusive purposes.

Substance abuse carries with it not only an important organic comorbidity, but also a marked socio-familial and economic repercussion.

**Disclosure of Interest:**

None Declared

